# Examining physiological, water relations, and hydraulic vulnerability traits to determine anisohydric and isohydric behavior in almond (*Prunus dulcis*) cultivars: Implications for selecting agronomic cultivars under changing climate

**DOI:** 10.3389/fpls.2022.974050

**Published:** 2022-08-25

**Authors:** Carolina Álvarez-Maldini, Manuel Acevedo, Daniela Estay, Fabián Aros, R. Kasten Dumroese, Simón Sandoval, Manuel Pinto

**Affiliations:** ^1^Instituto Ciencias Agroalimentarias Animales y Ambientales (ICA3), Campus Colchagua, Universidad de O′Higgins, San Fernando, Chile; ^2^Centro Tecnológico de la Planta Forestal, Instituto Forestal, Sede Biobío, San Pedro de la Paz, Chile; ^3^United States Department of Agriculture Forest Service, Rocky Mountain Research Station, Moscow, ID, United States; ^4^Laboratorio de Análisis y Modelamiento de Geoinformación, Departamento de Manejo de Bosques y Medio Ambiente, Facultad de Ciencias Forestales, Universidad de Concepción, Concepción, Chile

**Keywords:** drought, functional traits, xylem vulnerability to cavitation, hydroscapes, leaf water potential, stomatal conductance

## Abstract

The search for drought tolerant species or cultivars is important to address water scarcity caused by climate change in Mediterranean regions. The anisohydric–isohydric behavior concept has been widely used to describe stomatal regulation during drought, simply in terms of variation of minimal water potential (Ψ_min_) in relation to pre-dawn water potential (Ψ_pd_). However, its simplicity has sometimes failed to deliver consistent results in describing a complex behavior that results from the coordination of several plant functional traits. While *Prunus dulcis* (almond) is known as a drought tolerant species, little information is available regarding consistent metrics to discriminate among cultivars or the mechanisms underlying drought tolerance in almond. Here we show a sequence of plant stomatal, hydraulic, and wilting responses to drought in almonds, and the main differences between anisohydric and isohydric cultivars. In a pot desiccation experiment we observed that stomatal closure in *P. dulcis* is not driven by loss in turgor or onset of xylem cavitation, but instead, occurs early in response to decreasing Ψ_min_ that could be related to the protection of the integrity of the hydraulic system, independently of cultivar. Also, we report that anisohydric cultivars of *P. dulcis* are characterized by maximum stomatal conductance, lower water potentials for stomatal closure and turgor loss, and lower vulnerability to xylem cavitation, which are traits that correlated with metrics to discriminate anisohydric and isohydric behavior. Our results demonstrate that *P. dulcis* presents a strategy to avoid cavitation by closing stomata during the early stages of drought. Future research should also focus on below-ground hydraulic traits, which could trigger stomatal closure in almond.

## Introduction

The effects of climate change, such as an increase in temperature and altered patterns of frequency, distribution, and intensity of precipitation, have been extensively characterized (Cavin et al., 2013). Areas with Mediterranean climate, such as central Chile, are especially vulnerable to climate change because more frequent and extreme drought events are expected within the next decades (Schär et al., 2004; Bambach et al., 2013; del Pozo et al., 2019). Central Chile will experience a 20% decline in precipitation by 2050 coupled with an average annual increase of 3–4°C in temperature (Hannah et al., 2013), putting at risk the feasibility of cultivation of several agronomic species. Thus, drought leading to water stress in plants is the major factor reducing agricultural productivity (Chaves et al., 2003), which is one of the main economic activities of Mediterranean central Chile. To face drought in the long term, new suitable species and cultivars with drought tolerance characteristics are being identified and considered as options for the agricultural sector. One new, drought tolerant species being considered for central Chile is almond (*Prunus dulcis* L.) (Torrecillas et al., 1996), historically cultivated in other Mediterranean regions due to its capability to withstand water stress (Marsal et al., 1997; García-Tejero et al., 2018). Morphological and structural leaf adaptations that deliver protection against excessive water loss, such as reduced leaf area and stomatal density, thicker leaf cell walls, and increased cuticle thickness have been observed in almond cultivars (Camposeo et al., 2011; Oliveira et al., 2018). Physiologically, a gradual decrease in photosynthesis and stomatal conductance with increasing water stress is a characteristic response of drought-adapted plants and it has been frequently observed in almonds (Romero et al., 2004).

At a physiological level, drought is one of the most studied stresses in plants. The primary effect of drought is the reduction in stomatal conductance to decrease water losses by transpiration and preventing drops in leaf water potential (Ψ_L_), which in turn produces a reduction in carbon assimilation (Flexas et al., 2004, 2013), negatively affecting plant growth and crop production. Thus, stomatal regulation is key to control water loss and Ψ_L_ during water stress, but different species and cultivars have evolved other adaptive responses to limit dramatic decreases in Ψ_L_, leading to the isohydric and anisohydric behavior concept (Tardieu and Simonneau, 1998). In brief, isohydric species or cultivars maintain higher Ψ_L_ due to stomatal closure when the soil dries, whereas anisohydric species or cultivars sustain higher stomatal conductance allowing a drop in Ψ_L_ with the progression of water deficit (Tardieu et al., 1996). These behaviors do not represent a simple dichotomy but rather a continuum of strategies in stomatal control. This classification in the behavior of stomata during water stress has led to the search for anisohydric species or cultivars, which could hypothetically sustain higher carbon assimilation rates during drought, and it has been extensively studied in agronomically relevant species such as *Vitis vinifera* (Schultz, 2003; Soar et al., 2006; Prieto et al., 2010). On the contrary, in almond, the identification of drought-tolerant genotypes has only been focused on physiological changes in response to specific drought treatments, or limited to a narrow range in soil water potentials (Isaakidis et al., 2004; Rouhi et al., 2007; Yadollahi et al., 2011). This does not allow characterization of the stringency of stomatal control with respect to plant water status and the proper identification of anisohydric and isohydric cultivars.

Despite this very simplified classification of stomatal regulation during drought, it has been observed that the anisohydric or isohydric behavior of cultivars, and therefore their classification, depends largely on the soil water content and its hydraulic properties. Tramontini et al. (2014) showed that water relations of *V. vinifera* (Syrah, near-anisohydric, and Cabernet Sauvignon, near-isohydric) cultivars varied significantly depending on soil hydraulic conductivity, and Hochberg et al. (2018) and Domec and Johnson (2012) indicated that the shift between anisohydric and isohydric behavior depends on soil water potential where the plants water relation parameters are measured in relation to soil properties. Regardless of such inconsistencies in the definition of the anisohydric and isohydric behavior, some methodologies have been described to isolate the effect of environmental variation. The *hydroscape*, developed by Meinzer et al. (2016), describes the stringency of stomatal regulation of Ψ_L_ during the drying of soil. This concept incorporates ranges of pre-dawn water potential (Ψ_pd_) and mid-day minimum water potential (Ψ_min_) over which stomata are effective in controlling the drop of Ψ_L_ during soil desiccation until Ψ_pd_ = Ψ_min_ and describes a landscape where the plant is able to sustain photosynthesis until full stomatal closure. Thus, larger hydroscapes are indicative of a higher degree of anisohydry. This methodology has been previously assessed in almond rendering consistent results with other traits related to stomatal stringency during water stress (Álvarez-Maldini et al., 2021).

Hydroscapes have also been correlated with other plant functional traits linked to anisohydric behavior. The water potential at turgor loss point (Ψ_TLP_) is a strong predictor of an overall drought tolerance across species and biomes (Bartlett et al., 2012); a more negative Ψ_TLP_ is indicative of anisohydry and higher drought tolerance, a result initially reported by Meinzer et al. (2016) in different species. Likewise, through a meta-analysis, Fu and Meinzer (2018) found a strong correlation between the larger hydroscape areas and the lower Ψ_TLP_ values in species across different biomes. Also, Li et al. (2019) reported anisohydric *Eucalyptus* species with larger hydroscape areas were strongly linked to lower values of Ψ_TLP_, water potential at stomatal closure (P_gs90_), maximum stomatal conductance (gs_max_), and leaf and stem hydraulic vulnerability to embolism (PL_50_ and P_*x50*_). However, the hydroscape methodology, which has been mainly used to describe stomatal behavior of species distributed across various ecosystems, has yet to be tested broadly across a range of species and cultivars of agricultural interest and in relationship to other hydraulic and functional plant traits. Despite that Meinzer et al. (2016) and Li et al. (2019) have consistently shown a relationship between hydroscape area and traits such as Ψ_TLP_ in species from various environments, this link has yet to be proven among cultivars within a species. Given their reduced genetic variability, compared to different species adapted to distinct environments, changes in stomatal behavior and its relationship with other plant traits could be less clear. But Ψ_TLP_ and other functional traits have shown to be strong indicators of anisohydric behavior in *V. vinifera* (Tombesi et al., 2014; Albuquerque et al., 2020; Knipfer et al., 2020), which is in agreement with the multiple mechanisms that are integrated in the hydroscape that regulate plant water status and stomatal behavior.

Despite that this methodology has shown promise to properly characterize anisohydric and isohydric behavior, which in turn delivers information regarding cultivar selection, it is labor-intensive research to examine the regulation of water potential during prolonged periods of soil desiccation. Thus, correlating results from hydroscape analysis with informative functional traits such as Ψ_TLP_, gs_max_, or water potential at 50% loss of hydraulic conductivity (P50) would be less time-consuming and a valuable tool to help in the selection of cultivars adapted to drought prone environments. Also, identifying mechanisms and traits underlying drought resistance is essential to predict the future impacts of drought on productive species cultivated in the Mediterranean climate (Martin-StPaul et al., 2017). Considering the current need to assess almond cultivars for cultivation in central Chile, our objective was to describe anisohydric and isohydric behavior in almond cultivars. We used a pot desiccation experiment to explore dynamics of water potential, stomatal conductance, and hydroscape area toward better understanding the relationship of hydroscape area and plant functional traits.

## Materials and methods

### Plant material and growth conditions

The experiment was conducted between October of 2021 and February of 2022 at the experimental station in the Instituto de Ciencias Agroalimentarias Animales y Ambientales (ICA3) of the Universidad de O′Higgins, Chile (latitude: –34.61°, longitude: –70.99°, elevation: 352 m). The plant material corresponded to three cultivars of almond, Avijor (Ferragnès × Tuono), Isabelona (Blanquerna × Bella d′Aurons), and Soleta (Blanquerna × Bella d′Aurons), that were grafted onto rootpac-20 rootstocks (*Prunus besseyi* × *Prunus cerasifera*) 1 year earlier. Plants were donated by Agromillora Sur nursery (Río Claro, Maule region, Chile, latitude: –35.19°, longitude: –71.25°) in October of 2021 and transplanted into 7 L plastic pots (20 cm width × 20 cm length × 25 cm height) using a 1:1 v:v mixture of peat and perlite as substrate. An equal amount of substrate (by weight) was added to each pot (the oven-dry weight of substrate is used in Equation 1, below). The split-plot experiment had two irrigation treatments (WW and PD; whole plots) and three cultivars (Soleta, Isabelona, and Avijor; split plots). We had four blocks (each with an irrigation line) for each whole plot. Block corresponds to random effect, while cultivar and irrigation treatment are fixed effects. Each block had three experimental units (cultivars) and each experimental unit was composed by four pots with an individual plant. Thus, we employed 96 pots [2 irrigation treatments × 4 blocks × 3 cultivars × 4 pots (experimental units) of each cultivar]. Plants were irrigated daily by drip irrigation until they reached container capacity and maintained in this condition for acclimation for 1 month (November 2021) before onset of the irrigation treatments.

### Irrigation treatments

After the 1-month acclimation period, the two irrigation treatments commenced. The first irrigation treatment, well-watered (WW), maintained plants at container capacity for the remainder of the experiment. For the second irrigation treatment, pot desiccation (PD), irrigation ceased allowing for a progressive decrease in substrate water content due to plant transpiration (Álvarez-Maldini et al., 2021). To ensure that water losses corresponded to plant transpiration only and not to evaporation from the substrate, the surface of each pot was covered with a plastic film, allowing only the plant stem to emerge. Pot weights were subsequently recorded at midday for the remainder of the experiment. The gravimetric substrate water content (GWC, %) was calculated according to Equation 1:


(1) GWC(%)=[(P⁢o⁢t-P⁢o⁢tdry)P⁢o⁢twet-P⁢o⁢tdry]x 100

Where, *Pot* is the weight of the pot (plus substrate) at each measurement time, *Pot*_*dry*_ is the weight of the pot plus the initial, oven-dried weight of the substrate, and *Pot*_*wet*_ is the weight of the pot plus the substrate at container capacity.

### Midday and pre-down water potential and pressure-volume curves

Throughout application of the irrigation treatments, pre-dawn water potential (Ψ_pd_) and minimum mid-day water potential (Ψ_min_) were measured at least eight times for each cultivar since the beginning of the PD treatment, procuring water potential measurements along a wide range of GWC. For leaf water potential measurements, we randomly selected two plants per experimental unit and irrigation treatment (thus, 48 plants total; 2 plants × 2 irrigation treatments × 3 cultivars × 4 replicates). One fully developed leaf from the upper third of the canopy was excised with a razor blade and Ψ_L_ was measured with a Scholander pressure chamber model 1505D-EXP (PMS Instruments, Albany, OR, United States) (Scholander et al., 1965). The Ψ_pd_ was measured between 06:00 and 07:00 a.m., before dawn, and Ψ_min_ was measured between 13:00 and 14:00 p.m. local time, and both parameters were measured until Ψ_pd_ = Ψ_min_ was reached, which was indicative of the limits of stomatal control over plant water status.

Pressure volume (PV) curves were generated from plants of the WW treatment, for which one plant per experimental unit was randomly selected (4 plants total; 1 irrigation treatment × 3 cultivars × 1 plant of each cultivar). Leaf samples were collected before sunrise considering fully developed leaves from the upper third of the plant canopy and cut with a razor blade at the petiole and immediately stored in a sealed bag with damp paper and transported to the laboratory. Leaf PV curves were measured according to the protocol described by Tyree and Hammel (1972). Thus, leaves were allowed to slowly dehydrate under laboratory conditions, and Ψ_L_ and fresh mass were measured periodically with an analytical balance (Boeco model A0021E, Hamburg, Germany). The water potential at turgor loss point (Ψ_TLP_) was identified as the inflection point of the 1/Ψ_L_ vs. relative water content (RWC) curve. Mean modulus of elasticity (ε) was estimated as the slope of turgor potential (Ψ_p_) vs. RWC in the phase from full turgor to turgor loss point. Capacitance at full turgor (C_FT_) was calculated from the slope of the linear portion from RWC and Ψ_L_ before the Ψ_TLP_, normalized to saturated water content at leaf area.

### Gas exchange measurements

On the same days and plants that measurements of Ψ_pd_ and Ψ_min_ were recorded, gas exchange was measured at midday with a CIRAS-3 portable photosynthesis system equipped with a CFM-3 chlorophyll fluorescence module (PP Systems, Amesbury, MA, United States). The CO_2_ concentration in the leaf cuvette was adjusted to 400 ppm, the leaf temperature was maintained at 25 ± 1°C, and the PAR was set to 1,500 μmol photons m^–2^ s^–1^. Leaves were acclimated to cuvette conditions for at least 5 min before each measurement. Then, net photosynthesis (A_N_), stomatal conductance (gs), transpiration rate (E), and sub-stomatal CO_2_ concentration (Ci) were measured. The instantaneous water use efficiency (iWUE) was calculated as the ratio between A_N_ and E. The maximum photosynthetic and stomatal conductance rates (A_max_ and gs_max_, respectively) was considered as A_N_ and gs measured in plants before onset of the irrigation treatments.

### Stem vulnerability to xylem cavitation

Stem vulnerability curves were constructed using the air injection method with a double ended pressure sleeve connected to a Scholander pressure chamber (model described above). We used three plants of each cultivar following the protocol of Ennajeh et al. (2010). We procured the use of branches longer than 40 cm in length to accommodate maximum vessel length to avoid an open-vessel artifact (Cochard et al., 2013). Before the stem was inserted into the pressure-sleeve, the middle portion of bark was removed with a razor blade to facilitate air entry to xylem conduits. Leaves and side branches were removed, and cuts were sealed with glue and parafilm. A flexible tube connected to a solution tank filled with distilled water was connected to the basal end of the stem. The tank was installed 60 cm in height. Then, the stems were flushed at 0.06 MPa for 45 min to remove air bubbles and the maximum conductivity of the stem was measured (*K*_max_). Flow was measured gravimetrically by collecting the water from the distal end in a pre-weighed, 2-ml Eppendorf tube filled with cotton wool. Flow measurements were recorded for 2-min intervals.

After measuring *K*_max_, the pressure in the chamber was progressively increased, at 5-min intervals, to 0.5, 1.0, 1.5, 2.0, 2.5, 3.0, 3.5, 4.0, 5.0, 6.0, and 7.0 MPa. Stem hydraulic conductance (*K*_*h*_) was measured after slowly releasing the air and allowing the stem to equilibrate for 3-min, then flow measurements were recorded during a 2-min interval. The percentage loss of conductivity (PLC) was calculated using Equation 2 as follows:


(2) $$PLC\left(\%\right)=100x\left(1-\frac{K_{h}}{K_{\text{max}}}\right)$$

The relationship between stem hydraulic conductance and PLC was fitted with a Weibull curve using the *fitplc* package (Duursma and Choat, 2017) in the R software (version 4.2.0, R Core Team, 2022). The water potential corresponding to a 50% loss in conductivity (P50) and bootstrapped confidence intervals (CI) were calculated.

### Metrics to determine anisohydric and isohydric behavior

Considering leaf water relations and gas exchange measurements described above, different metrics to assess anisohydric and isohydric behavior were calculated.

First, the slope of the relationship between Ψ_pd_ and Ψ_min_ (σ) was calculating using linear regression according to the methodology described by Martínez-Vilalta et al. (2014).

Second, the hydroscape area (hereafter, hydroscape) was measured following methodology described in Meinzer et al. (2016) and Álvarez-Maldini et al. (2021). In brief, the hydroscape is the area comprising the Ψ_pd_ vs. Ψ_min_ regression line and a 1:1 line, which is calculated according to Equation 3:


(3) $$H\,y\,d\,r\,o\,s\,c\,a\,p\,e=\frac{\left(a\,x\,b\right)}{2}$$

Where *a* is the intercept of the Ψ_pd_ vs. Ψ_min_ regression line, representing the most negative Ψ_min_ when Ψ_pd_ = 0, and *b* is the intersection of Ψ_pd_ vs. Ψ_min_ and the 1:1 line, corresponding to the water potential at Ψ_pd_ = Ψ_min_ which indicates the limit of stomatal control to prevent further decrease in leaf water potential.

Third, the water potential at stomatal closure (Ψ_gs90_) was calculated according to Li et al. (2019). Briefly, the stomatal conductance (gs, described in section “Gas exchange measurements”) was plotted against Ψ_min_ and fitted with a weighted polynomial regression to obtain Ψ_gs90_ using the *fitplc* package from R software (version 4.2.0) (Duursma and Choat, 2017).

### Statistical analysis

To assess the relationship between Ψ_pd_ and Ψ_min_, a first-order kinetic model was fitted using PROC REG procedure (SAS Institute Inc., Cary, NC, United States) (Álvarez-Maldini et al., 2021).

Cultivar effects on A_max_, gs_max_, E_max_, iWUE_max_, GWC, π_o_, Ψ_TLP_, RWC_TLP_, ε, and C_FT_ were assessed using one-way analyses of variance using generalized mixed models using procedure PROC GLIMMIX (SAS Institute Inc., Cary, NC, United States) with selection of distribution considering the Akaike Information Criteria. Differences among means were determined using a Tukey (HSD) test for multiple comparisons.

To evaluate the effect of cultivar and irrigation treatment on A_N_, E, gs, iWUE, Ψ_min_ and Ψ_pd_ an ANOVA analysis was performed according to our experimental design as described above. Differences among means were determined by using a Tukey (HSD) test for multiple comparisons.

To assess the relationship between gs and Ψ_min_ during PD, a first-order kinetic model was fitted using the PROC NLIN procedure (SAS Institute Inc., Cary, NC, United States) with the Gauss-Newton method through a derivative-free algorithm. The cultivar effect was evaluated using the extra sums of squares principle (Bergerud, 1996).

Estimation and inference of regression with piecewise linear model was used to perform the analysis of the water potential (WP) curve. The model generates a linear estimate and calculates the boundaries between linear phases and corresponding slope values according to the methodology described by Muggeo (2003) and adapted by Muggeo et al. (2014) to generate models by different segments with continuous intersection point and with unequal slopes. In our analysis we generated three segments to intersection points or boundaries (Θ_1_ and Θ_2_). All the parameters for the model were fitted using a least-squares solver implemented in R software (R Core Team) by “*segmented*” package v1.6-0 (Muggeo, 2022).

All visualizations were made using SigmaPlot 14 (Systat Software Inc., San Jose, CA, United States).

## Results

### Initial plant-water relations and gas exchange among cultivars

At the end of the acclimation period and before onset of the irrigation treatments, significant differences were observed in maximum values in the gas exchange parameters of gs_max_, E_max_, and iWUE_max_ among cultivars, while no differences were observed in A_max_ (Table 1). Soleta presented a significantly higher gs_max_, followed by Isabelona, and lastly Avijor; a similar trend was observed in E_max_, with Soleta and Isabelona reaching significantly higher values, followed by Avijor. Consistently as consequence of higher stomatal conductance, Soleta and Isabelona displayed significantly lower iWUE_max_, and Avijor with significantly higher iWUE_max_ (Table 1).

**TABLE 1:** Mean (± standard error) values of leaf photosynthetic traits of *Prunus dulcis* cultivars measured before the imposition of the pot desiccation treatment.

Cultivar	Traits			
	
	A_max_ (μmol CO_2_ m^–2^ s^–1^)	gs_max_ (mmol H_2_O m^–2^ s^–1^)	E_max_ (mmol H_2_O m^–2^ s^–1^)	iWUE_max_ (mmol CO_2_ mol^–1^ H_2_O)
Avijor	12.24 ± 0.67 ns	363.32 ± 24.14 **c**	5.18 ± 0.22 **b**	2.39 ± 0.13 **a**
Isabelona	11.24 ± 0.88 ns	517.39 ± 19.95 **b**	6.31 ± 0.13 **a**	1.82 ± 0.16 **b**
Soleta	13.22 ± 0.90 ns	623.36 ± 27.60 **a**	6.83 ± 0.17 **a**	1.97 ± 0.14 **b**

A_max_, maximal photosynthetic rate on well-watered plants; gs_max_, maximum stomatal conductance on well-watered plants; E_max_, maximum transpiration rate on well-watered plants; iWUE_max_, maximum water use efficiency on well-watered plants. Different letters indicate significant differences among cultivars at *p* ≤ 0.05 according to Tukey test.

At the onset of the irrigation treatments the pot water content of Avijor, Isabelona, and Soleta was similar (102.3 ± 1.4%, 99.8 ± 4.0%, and 100.2 ± 2.1%, respectively).

### Hydraulic traits of cultivars

In relation to the pressure-volume curve analysis, cultivar was significant for Ψ_TLP_ (Table 2; Soleta < Avijor = Isabelona) but not for SWC, π_*o*_, RWC_TLP_, C_FT_, or ε traits. We observed a trend (*p* = 0.0791) for higher πo in Soleta, followed by Isabelona, and then Avijor.

**TABLE 2:** Values of key metrics to describe anisohydric and isohydric behavior in *Prunus dulcis* cultivars and of hydraulic traits derived from pressure volume curves (mean ± standard deviation), stem vulnerability curves (mean, CI in brackets) and leaf water potential causing 90% of stomatal closure (Ψ_gs90_) for different almond cultivars.

Cultivar	σ (MPa MPa^–1^)	Hydroscape (MPa^2^)	SWC	π_*o*_ (MPa)	Ψ_TLP_ (MPa)	RWC_TLP_	ε (MPa)	C_FT_ (mol m^–2^ MPa^–1^)	Ψ_gs90_ (MPa)	P50 (MPa)
Avijor	0.922	8.74	2.43 ± 0.28	1.52 ± 0.42	–2.01 ± 0.44 **a**	88.26 ± 1.52	12.91 ± 4.87	0.59 ± 0.09	–1.72 [1.69, 2.91]	–2.97 [2.53, 3.46]
Isabelona	0.811	8.13	2.68 ± 0.82	1.62 ± 0.25	–2.31 ± 0.13 **a**	83.08 ± 3.50	11.38 ± 5.74	0.80 ± 0.30	–2.25 [2.04, 2.53]	–3.80 [3.41, 4.28]
Soleta	0.867	9.23	2.05 ± 0.66	1.97 ± 0.24	–2.87 ± 0.17 **b**	64.22 ± 3.24	11.85 ± 2.14	0.77 ± 0.14	–2.14 [1.99, 2.33]	–3.73 [3.47, 4.02]
*p*-value	–	–	0.3530	0.07916	**0.0059**	0.2338	0.8885	0.3459	–	–

σ, slope of the relationship between Ψ_pd_ and Ψ_min_; SWC, saturated water content; π_o_, osmotic potential at full turgor; Ψ_TLP_, water potential at turgor loss point; RWC_TLP_, relative water content at turgor loss point; ε, modulus of elasticity; and C_FT_, capacitance at full turgor. P50, water potential at 50% loss of hydraulic conductivity. Different letters within each trait indicate significant differences among means (*p* ≤ 0.05). Bold values indicate significant differences between cultivars.

Water potential at 50% loss of conductivity (P50) showed different values among cultivars. Soleta and Isabelona had similar P50 values (–3.73 MPa and –3.80 MPa, respectively) that were lower than Avijor (–2.97 MPa) (Table 2).

### Different behavior of cultivars during pot desiccation

The PD experiment lasted 50 days between December of 2021 until January of 2022. Water potential (Ψ_min_ and Ψ_pd_) and gas exchange parameters were measured 1, 9, 15, 20, 23, 27, 35, 44, and 50 days after the beginning of the PD treatment. During the PD treatment imposition, GWC content decreased steadily, reaching pot water contents of 35.9 ± 3.7%, 31.9 ± 1.9%, and 30.7 ± 4.8% in Avijor, Isabelona, and Soleta, respectively. Pot water content in the WW treatment was 89.6 ± 5.8% in Avijor, 90.0 ± 1.3% in Isabelona, and 86.6 ± 4.8% in Soleta.

Among cultivars, all leaf water relations and gas exchange parameters were affected by the PD treatment. At conclusion of the PD treatment, A_*N*_ was significantly affected by cultivar (*p* < 0.0001), and iWUE was only affected by the PD treatment (*p* = 0.0277) with significantly lower values in stressed plants. Minimum water potential was independently affected by the irrigation treatment and cultivar, while both factors significantly interacted to affect E, gs and Ψ_pd_ (Table 3). At the end of the PD treatment and for all cultivars, Ψ_min_ decreased significantly compared with the WW treatment, reaching minimum values in Soleta followed by Avijor and by Isabelona. In all the parameters affected by the irrigation × cultivar interaction (E, gs and Ψ_pd_), the PD treatment significantly decreased values compared to the WW treatment. In regard to gs, at the end of the PD treatment, Isabelona reached the lowest value, with higher gs in Soleta. Transpiration rate (E) was higher in Soleta in the WW treatment compared with Avijor and Isabelona, but no differences among cultivars were observed in the PD treatment (Table 3). The lowest values of Ψ_pd_ at the end of the PD treatment for Avijor, Isabelona, and Soleta were –2.93 ± 0.29, –2.39 ± 0.29, and –3.38 ± 0.12 MPa, respectively (Table 3).

**TABLE 3:** Mean (± standard deviation) values of leaf photosynthetic traits and water potential at conclusion of the pot desiccation (PD treatment), source of variation, and *p*-values of *Prunus dulcis* cultivars subjected to PD and well-watered (WW) irrigation treatments.

	iWUE (mmol CO_2_ mol^–1^ H_2_O)	Ψ_min_ (MPa)	A_*N*_ (μmol CO_2_ m^–2^s^–1^)	E (mmol H_2_O m^–2^s^–1^)		gs (mmol H_2_O m^–2^s^–1^)		Ψ_pd_ (MPa)	
						
				WW	PD	WW	PD	WW	PD
**Cultivar (C)**									
Avijor	–	–2.94 ± 0.43 **ab**	8.44 ± 2.31 **a**	5.33 ± 0.15 **b**	1.14 ± 0.12 **c**	374.68 ± 19.59 **b**	51.00 ± 6.08 **cd**	–0.70 ± 0.03 **a**	–2.93 ± 0.29 **d**
Isabelona	–	–2.83 ± 0.44 **a**	6.16 ± 1.70 **b**	6.45 ± 0.18 **a**	0.95 ± 0.07 **c**	530.95 ± 27.77 **a**	41.80 ± 3.18 **d**	–1.00 ± 0.09 **b**	–2.39 ± 0.29 **c**
Soleta	–	–3.24 ± 0.49 **b**	7.26 ± 1.91 **ab**	6.30 ± 0.06 **a**	1.19 ± 0.07 **c**	531.85 ± 20.21 **a**	53.83 ± 3.53 **c**	–0.95 ± 0.05 **b**	–3.38 ± 0.12 **e**
Treatment (T)									
WW	2.06 ± 0.18 **b**	–1.83 ± 0.08 **a**	–	–	–	–	–	–	–
PD	2.18 ± 0.12 **a**	–4.18 ± 0.10 **b**	–	–	–	–	–	–	–
Source of variation									
Block	0.3920	0.3267	0.6810	0.2318		0.8034		0.2007	
C	0.3865	**<0.0001**	**<0.0001**	<0.0001		<0.0001		<0.0001	
T	**0.0277**	**0.0002**	0.0624	<0.0001		0.0370		<0.0001	
C *x* T	0.1456	0.8410	0.2343	**0.0003**		**0.0084**		**<0.0001**	

gs, stomatal conductance; Ψ_min_, minimum midday water potential; A_N_, net photosynthesis; E, transpiration rate; WUE, water use efficiency; Ψ_pd_, pre-dawn water potential. Different letters within columns indicate statistical differences among means (*p* ≤ 0.05). Bold values indicate significant differences between cultivars.

**FIGURE 1:**
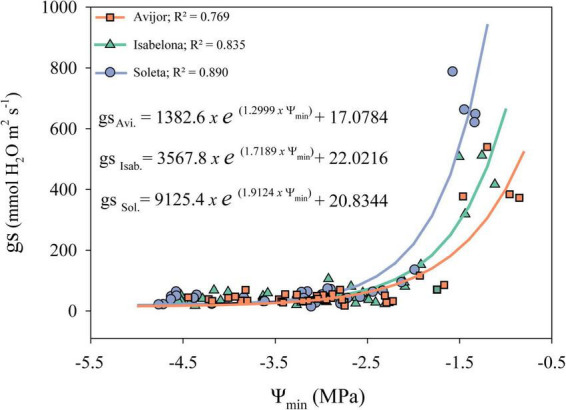
Effect of midday minimum water potential (Ψ_min_) on stomatal conductance (gs) during the pot desiccation treatment of *Prunus dulcis* cultivars. Symbols indicate measured data and lines correspond modeled data for each cultivar. Fitted models and corresponding *R*^2^-value for all cultivars are shown in the panel.

Although, as previously described, the PD treatment affected leaf water potential and gas exchange parameters at the end of the experiment, the desiccation treatment influenced the behavior of stomatal conductance differently, with decreasing Ψ_min_ of each cultivar during the first stages of the desiccation treatment (Figure 1). Thus, different models were fitted for Avijor, Isabelona, and Soleta. A faster decrease in gs was observed in Avijor at higher Ψ_min_ values. On the contrary, Soleta sustained higher stomatal conductance for a longer period (at more negative Ψ_min_) during the progression of PD, while Isabelona displayed an intermediate behavior between Soleta and Avijor (Figure 1). This pattern was similar for water potential at stomatal closure (Ψ_*gs90*_) (Table 2), with Soleta and Isabelona displaying lower values of Ψ_*gs90*_ (–2.14 MPa and –2.25 MPa, respectively) and Avijor displaying a higher value (–1.72 MPa), although the large confidence intervals of Avijor overlapped with the Ψ_*gs90*_ of the other two cultivars.

Regarding the metrics of stringency of stomatal control, the slope of the linear regression fitted to the relationship of Ψ_pd_ vs. Ψ_min_ (σ) revealed that σ only ranged between 0.811 MPa MPa^–1^ in Isabelona and 0.922 MPa MPa^–1^ in Avijor (Table 2), with none of the cultivars displaying a perfect isohydric or anisohydric behavior (slopes 0 or 1, respectively).

**FIGURE 2:**
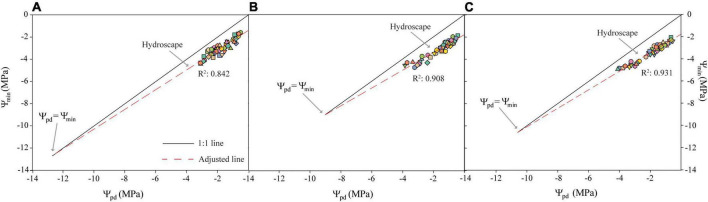
Trajectories of pre-dawn water potential (Ψ_pd_) vs. minimum midday water potential (Ψ_min_) during the pot desiccation treatment for the *Prunus dulcis* cultivars Avijor **(A)**, Isabelona **(B)**, and Soleta **(C)**. Solid black line represents the 1:1 line, dashed red line is the fitted linear regression of measured data. Different symbols in colors represent different individual plants measured. Hydroscape is represented by the area bounded by the regression and the 1:1 line.

Despite not observing perfect anisohydric or isohydric behavior, the hydroscape area ranged from 8.74 MPa^2^ in Avijor to 9.223 MPa^2^ in Soleta (Figure 2 and Table 2), with Isabelona displaying an intermediate value (Table 2). Thus, cultivar rankings based on hydroscape area from the more isohydric to the more anisohydric is: Isabelona, Avijor, and Soleta.

**FIGURE 3:**
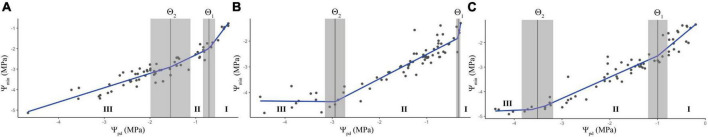
Adjustment of the piecewise linear regression (PLR) model on the relationship between pre-dawn water potential (Ψ_pd_) and minimum mid-day water potential (Ψ_min_) on the *Prunus dulcis* cultivars Avijor **(A)**, Isabelona **(B)**, and Soleta **(C)**. Black circles indicate data from each individual plant, blue line is the fitted model. Vertical solid lines are boundaries between phases I and II (Θ_1_) and between phases II and III (Θ_2_), corresponding SE values are displayed in gray lines. Model output parameters are summarized in Table 4.

As previously demonstrated by Knipfer et al. (2020), the relationship between Ψ_pd_ and Ψ_min_ during drought is not linear, and is constituted by three phases of plant dehydration with break points between each phase that are indicative of the thresholds of stomatal closure and leaf turgor loss. Accordingly, we were able to perform the analysis of the water potential curve (WP curve) during pot desiccation for each cultivar and identify three distinct phases along the WP curve (Figure 3 and Table 4), although it displayed a different shape than the one described by Knipfer et al. (2020). According to the slope of the three phases (Table 4), a steeper decrease in water potential was observed during the first stages of the PD treatment, which continued until –0.803 MPa in Avijor, –0.334 MPa in Isabelona, and –0.997 MPa in Soleta, corresponding to the boundary between the first and second phase (Θ_1_). Following Θ_1_, reductions in water potential were less pronounced in the second and third phases, observed through lower slope values (β_2_ and β_3_) (Table 4). The boundary between the second and the third phase (Θ_2_) was higher in Avijor (–1.553 MPa), Isabelona (–2.941 MPa), and Soleta (–3.513 MPa) (Figure 3).

**TABLE 4:** Summary of output parameters from the piecewise linear regression (PLR) model as described by Knipfer et al. (2020) used for the analysis between Ψ_pd_ and Ψ_min_ for *Prunus dulcis* cultivars.

Cultivar	Θ_1_ (MPa)	Θ_2_ (MPa)	β_1_	β_2_	β_3_	RMSE	*R* ^2^
Avijor	–0.803	–1.553	2.594	1.015	0.711	0.274	0.9135
Isabelona	–0.334	–2.941	11.492	0.948	–0.018	0.319	0.8772
Soleta	–0.997	–3.513	1.610	0.865	0.078	0.305	0.9182

Θ_1_, boundary between phases I and II; Θ_2_, boundary between phases II and III; β_1_, slope of phase I; β_2_, slope of phase II; β_3_, slope of phase III; R^2^, fitted; and RMSE, root mean square deviation.

## Discussion

The anisohydric and isohydric concept has been used for several decades as a framework to describe different behaviors of plant water-relations during drought. However, this concept is not exempt from criticism due to common lack in consistency, with species or cultivars shifting from isohydric to anisohydric behavior, and vice versa (Hochberg et al., 2018). Isohydric and anisohydric behavior are simply described in terms of changes in Ψ_min_ in response to Ψ_pd_, although it represents a whole-plant hydraulic strategy resulting from the coordination and trade-offs among different plant functional traits (Meinzer et al., 2017; Fu et al., 2019; Henry et al., 2019). Thus, using plant functional traits to predict species distribution, dynamics, and responses to environmental change has been important in physiological ecology (Adier et al., 2014), but is now rapidly gaining usefulness in the search for cultivars of interest in the agricultural industry. To fully comprehend the behavior in stomatal regulation during water stress of three almond cultivars, we assessed several metrics of anisohydric and isohydric behavior and leaf and stem functional traits for a more comprehensive framework to characterize plant response to drought (Skelton et al., 2015).

Initially, we assessed the slope of the Ψ_min_ vs. Ψ_pd_ (σ) as previously described by Martínez-Vilalta et al. (2014), but none of the cultivars displayed neither perfect isohydric nor anisohydric behavior (0 or 1 values, respectively). Although σ showed a tendency of Avijor to have a more isohydric behavior in this study, it has failed to correlate with other metrics of stomatal sensitivity in 44 species (Martínez-Vilalta and Garcia-Forner, 2017) and it has proven to be inconsistent to rank species/cultivars along a continuum of isohydric to anisohydric depending of the values of Ψ_pd_ (Poni et al., 2007; Domec and Johnson, 2012; Hochberg et al., 2018). We also used the hydroscape metric to assess the anisohydric and isohydric behavior of the three almond cultivars. According to Meinzer et al. (2016), the hydroscapes describe a water potential landscape over which plants operate before drought-induced stomatal closure, with anisohydry characterized by larger areas. Our results regarding hydroscape values are similar to the more anisohydric species reported by Meinzer et al. (2016) with 8.4 MPa^2^ in *Quercus garryana*, and higher to the values reported by Li et al. (2019) in *Eucalyptus* species. Thus, according to this metric the rank of our cultivars from more isohydric to more anisohydric is Avijor, Isabelona, and Soleta. Despite the above-mentioned issues of σ, both metrics (hydroscapes and σ) ranked the cultivars equally. These consistent results could be related to the wide range in Ψ_pd_ we measured. A wide range of hydroscape areas have been reported among different species (Meinzer et al., 2016; Li et al., 2019), while in this study a lower range in area variation among cultivars (8.13 MPa^2^ vs. 9.23 MPa^2^) was observed. Regardless of this lower variation, cultivars also displayed differential behavior observed in stomatal responses to decreasing water availability (Figure 1). While Avijor had a faster decrease in stomatal conductance with decreasing Ψ_min_, Soleta sustained higher stomatal conductance at lower values of Ψ_min_; this points toward an isohydric behavior in Avijor and an anisohydric behavior in Soleta. This agrees with Tombesi et al. (2014) who reported stomatal closure at higher leaf and stem water potentials in a near-isohydric *V. vinifera* cultivar (Montepulciano) vs. the anisohydric cultivar (Sangiovese).

Despite reports that species with higher sensitivity to stomatal closure during leaf dehydration also have higher values of gs_max_ (Skelton et al., 2015; Henry et al., 2019), our results showed that the more anisohydric cultivar Soleta, which sustained higher gs at lower Ψ_min_, also presented higher gs_max_, E_max_, and lower iWUE (Table 1) under conditions of high water availability. This concurs with Meinzer et al. (2017), who reported that anisohydric species have faster kinetics of stomatal opening and activation of photosynthesis with greater stomatal conductance, photosynthetic capacity, and lower iWUE, characteristic of species from dry regions that aim to maximize the utilization of unpredictable and rare precipitation events (Li et al., 2016, 2018). This trait has also been observed between anisohydric and isohydric *Vitis* cultivars (Dayer et al., 2020). These results indicate that anisohydric cultivars such as Soleta are characterized by physiological traits linked to drought tolerant species that maximize carbon gain during periods of high water availability.

As expected, the imposition of the PD experiment caused an increase in iWUE in all cultivars at the beginning of the experiment (Supplementary Figure 1). However, during the first 30 days of the PD treatment, the more anisohydric cultivars Soleta and Isabelona sustained higher iWUE than the isohydric-behaving Avijor (Supplementary Figure 1). Higher iWUE during PD in Soleta and Isabelona are explained mainly by maintenance of higher photosynthesis rates than Avijor during PD. Although it has been previously reported than isohydric cultivars are expected to present higher iWUE (Schultz, 1996; Poni et al., 2007), our results agree with Pou et al. (2012) who indicates that anisohydric *Vitis* cultivars show higher WUE due to maintenance of photosynthesis stability.

The water potential at stomatal closure (Ψ_gs90_) is also a trait that can accurately quantify anisohydric and isohydric behavior (Skelton et al., 2015; Hochberg et al., 2018), where a more negative Ψ_gs90_ indicates that plants can sustain carbon assimilation over a wider range of leaf water potentials, which is in direct relationship with larger hydroscape areas. In agreement, we observed that the more anisohydric cultivars Soleta and Isabelona had lower Ψ_gs90_, in contrast with the more isohydric cultivar Avijor (Table 2); a similar trend was also observed in Ψ_TLP_ values among cultivars. The water potential at turgor loss point has been widely used as an indicator of drought tolerance, as leaves with lower Ψ_TLP_ maintain open stomata, hydraulic conductance, and are able to grow under dryer conditions (Bartlett et al., 2012; Scoffoni et al., 2012; Trifilò et al., 2015). Recently, lower Ψ_TLP_ values have been positively correlated with larger hydroscapes areas, and thus anisohydry (Meinzer et al., 2016; Li et al., 2019), which supports our results regarding the contrasting anisohydric behavior of Soleta and the isohydric behavior of Avijor. Our values of Ψ_TLP_ and Ψ_gs90_ were also similar to the ones reported by Hernandez-Santana et al. (2016) in almond (–2.26 and –2.14 MPa, respectively), which are in the range of values displayed by drought-tolerant species.

Although no significant differences were observed in π_0_ among cultivars (*p* = 0.0791) (Table 2), the trend in values with higher π_0_ in Soleta and lower in Avijor, and the absolute lack of differences in ε, support the notion that contrast observed in Ψ_TLP_ among cultivars is mainly driven by osmotic adjustment and not by modifications of mechanical properties of the cell wall. This is consistent with a previous meta-analysis (Bartlett et al., 2012) indicating that π_0_ is the main driver of Ψ_TLP_ across species, and that ε does not play a role in determining drought-tolerance. Regardless of the relationship between Ψ_TLP_ and Ψ_gs90_, our results suggest that Ψ_TLP_ is not the main factor triggering stomatal closure because, independently of the cultivar, complete stomatal closure occurred before turgor loss (Ψ_gs90_ > Ψ_TLP_). This indicates that stomatal closure is uncoupled from bulk leaf water status. Similar results were reported in anisohydric and isohydric *Vitis* cultivars (Tombesi et al., 2014) and several woody species adapted to sites with contrasting resource availability (Henry et al., 2019).

Consistent with the previously described traits, the P50 displayed that same variation among almond cultivars, with more negative values in Soleta and Isabelona than in Avijor (Table 2), indicating a higher vulnerability to cavitation in Avijor. Information regarding the relationship between vulnerability to cavitation and anisohydric and isohydric behavior is scarce for cultivars, but Alsina et al. (2007) reported large variability in PCL_50_ across *Vitis* cultivars (–1 to –3 MPa). Reports in different species have found a correlation between a higher xylem vulnerability to cavitation with isohydric behavior, such as in *Acer pseudoplatanus* and *Corylus avellana* (Li et al., 2016). It also has been proposed that xylem cavitation is the hydraulic signal that triggers stomatal closure in several species (Salleo et al., 2000; Nardini et al., 2001; Zufferey et al., 2011). On the contrary, the differences between Ψ_gs90_ and Ψ_TLP_ in our results showed that stomatal closure occurred before the 50% loss of xylem hydraulic conductivity in the three cultivars (Ψ_gs90_ > P50). Recent evidence also supports the notion that stomatal closure precedes xylem cavitation at community and global scales (Klein, 2014; Bartlett et al., 2016; Martin-StPaul et al., 2017) and even among cultivars (Carminati and Javaux, 2020; Dayer et al., 2020; Gambetta et al., 2020). These results indicate that the hydraulic safety margin (HSM), defined as the remainder of Ψ_gs90_ minus P_50_, is generally positive and thus constitutes a strategy to avoid lethal embolism.

As another metric to describe the dynamics of leaf water potential during pot desiccation, we performed an analysis of the water potential curve using a piecewise linear regression according to Knipfer et al. (2018). Here, authors describe the triphasic nature of the WP curve and the prediction of the first boundary (Θ_1_) between phases I and II that would correspond to the threshold at which leaf gas exchange is substantially decreased, and the second boundary (Θ_2_) matching the Ψ_TLP_. We were able to model the three phases of the WP curve and predict its corresponding boundaries and slopes (Table 4), but the shape of our curve diverged from the one described by Knipfer et al. (2020). Specially, a steeper decrease in water potential was observed in phase II in our cultivars while Knipfer et al. (2020) showed a minor reduction in Ψ_min_ with the continuous decrease in Ψ_pd_. A faster dry-down in our experiment could explain the steeper reduction in Ψ_min_ during the PD treatment, indicating that factors that affect the rates of water loss and transpiration also influence dynamics of water potential. Also, the almond experiment described by Knipfer et al. (2020) was performed in 56-L potted plants, which can affect the pace of the dry-down, and thus, the dynamics of water potential, explaining the steeper decline in Ψ_min_ during phase II of our experiment. Differences in cultivar/rootstock combination could also affect the shape of the WP curve. Our predicted boundaries, Θ_1_ and Θ_2_, did not match stomatal closure nor Ψ_TLP_, respectively, as indicated by Knipfer et al. (2020). It is interesting to note, however, the smaller distance between Θ_1_ and Θ_2_ boundaries in the isohydric Avijor (Figure 3A), compared to the larger distance described between the two boundaries in the more anisohydric cultivars Isabelona and Soleta (Figures 3B,C). Along the three phases of the WP curve, a consistent reduction in the slope of each phase was observed (Table 4), thus the faster reduction in Ψ_min_ occurred during the initial stages of the PD treatment until Θ_1_, which could have triggered the onset of stomatal closure.

**FIGURE 4:**
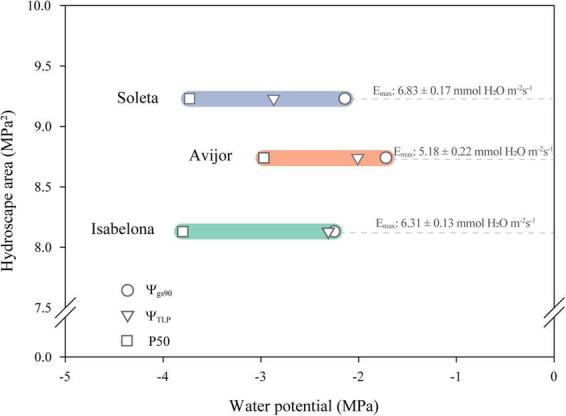
Relationships among hydroscape area and traits linked to anisohydric and isohydric behavior in the *Prunus dulcis* cultivars Avijor Isabelona, and Soleta. Ψ_gs90_: leaf water potential causing 90% of stomatal closure, Ψ_TLP_: water potential at turgor loss point, and P50: water potential at 50% loss of hydraulic conductivity. Values of maximum transpiration rate (E_max_) are shown for each cultivar.

Considering all the traits described above, we observed responses to the PD treatment that suggest almond has an avoidance strategy rather than a tolerance strategy for hydraulic failure as described by Hochberg et al. (2017). First, the steep decrease in Ψ_min_ in response to decrease in Ψ_pd_ (Figure 1) eventually led to stomatal closure (Ψ_gs90_) at water potentials higher than the loss of cell turgor (Ψ_TLP_) and preceded development of cavitation in xylem conduits (P50) (Figure 4). This concurs with recent research stating that drought-resistant plants close their stomata at water potentials higher than that at which substantial embolism occurs (Martin-StPaul et al., 2017). Second, we observed leaf shedding in all cultivars, especially Avijor, which further supports an avoidance strategy toward protection of perennial tissues. These responses did, however, prevent us from observing a direct measurement of Ψ_min_ = Ψ_pd_. Thus, for hydroscape calculation, Ψ_min_ = Ψ_pd_ was estimated by extrapolation of water potential values at the end of the PD experiment, rendering unreliable Ψ_min_ = Ψ_pd_ values well beyond limits of stomatal control. Then, because the WP curves did not follow a strictly linear fit (Table 4 and Figure 3), we estimated Ψ_min_ = Ψ_pd_ as the intersection between a 1:1 line and the modeled piecewise regression, which yielded Ψ_min_ = Ψ_pd_ values for Avijor (–6.15 MPa), Isabelona (–4.33 MPa), and Soleta (–4.83 MPa) that were closer to the observed water potential values for turgor loss and loss of hydraulic conductivity.

Despite the above-mentioned issues when calculating hydroscapes, a clear relationship was observed between hydroscape area and traits related to anisohydric behavior. Thus, Soleta cultivar with a near-anisohydric behavior displayed larger hydroscape area with lower values of Ψ_gs90_, Ψ_TLP_, and P50, contrary to the behavior displayed by Avijor (Figure 4). Thus, considering the above-mentioned traits for Soleta and Isabelona with an increased iWUE during the first stages of drought indicate, both cultivars are suitable candidates to be established under Mediterranean climate conditions of central Chile, especially in areas with restricted access to irrigation water.

Furthermore, our results regarding Soleta cultivar are in agreement with Álvarez et al. (2020) who indicated that this cultivar displayed physiological responses linked to drought tolerance when growing with its original root system (self-rooted). This result was, however, diminished when plants were grafted onto the dwarfing rootstock Rootpac-20. These results correlate with Ben Yahmed et al. (2016) that described a poor adaptation of this rootstock to Mediterranean or rain-fed conditions. Thus, considering these reports, it is important to highlight the influence of the rootstock on drought tolerance, and that the anisohydric behavior of Soleta and Isabelona cultivars may have been attenuated by the Rootpac-20 rootstock. Future work should focus on the effects of rootstock on anisohydric and isohydric behavior and consider the use of self-rooted plants for cultivation under Mediterranean conditions.

## Conclusion

Our results from a pot desiccation treatment revealed that almond cultivars followed a similar response to drought: decreasing Ψ_min_ led to stomatal closure to avoid hydraulic disfunction. The different thresholds of water potentials indicate that neither turgor loss nor xylem cavitation are the hydraulic signal to induce stomatal closure, but rather that loss in soil hydraulic conductivity could be the main trigger of stomatal closure. Thus, future research regarding almond and the differences in anisohydric and isohydric behavior among its cultivars should account for below- and above-ground hydraulic traits.

Cultivars did, however, display differences in anisohydric and isohydric behavior. Anisohydric cultivars, Soleta and Isabelona, displayed larger hydroscape areas. Moreover, they presented different boundaries along the WP curve, higher gs_max_ and sustained higher stomatal conductance at lower Ψ_min_, lower Ψ_TLP_ and vulnerability to xylem cavitation (P50), and higher iWUE than the isohydric cultivar Avijor. Because Ψ_TLP_, P50, and gs_max_ are easily measured in well-watered plants, their assessment could be readily used to discriminate anisohydric and isohydric plants under consideration for agronomic development in Mediterranean climates without the laborious need for controlled drought experiments. Selecting plants more anisohydric in nature, such as Soleta in this research, with their ability to withstand cavitation better than their isohydric cohorts, may be preferred given expected changes in Mediterranean climates.

## Data availability statement

The raw data supporting the conclusions of this article will be made available by the authors, without undue reservation.

## Author contributions

CÁ-M, MP, and MA conceived and designed the experiment. CÁ-M, DE, and FA performed physiological measurements. MA and SS: contributed to data analysis. CÁ-M and RD wrote the manuscript. All authors contributed to the article and approved the submitted version.
