# Supplementary material for: Examining physiological, water relations, and hydraulic vulnerability traits to determine anisohydric and isohydric behavior in almond (Prunus dulcis) cultivars: Implications for selecting agronomic cultivars under changing climate
Source: Front Plant Sci. 2022 Aug 25;13:974050. doi: 10.3389/fpls.2022.974050 (PMC9453546; doi:10.3389/fpls.2022.974050)
Supplement: Supplementary file 1 [file Image_1.pdf]

Supplementary information

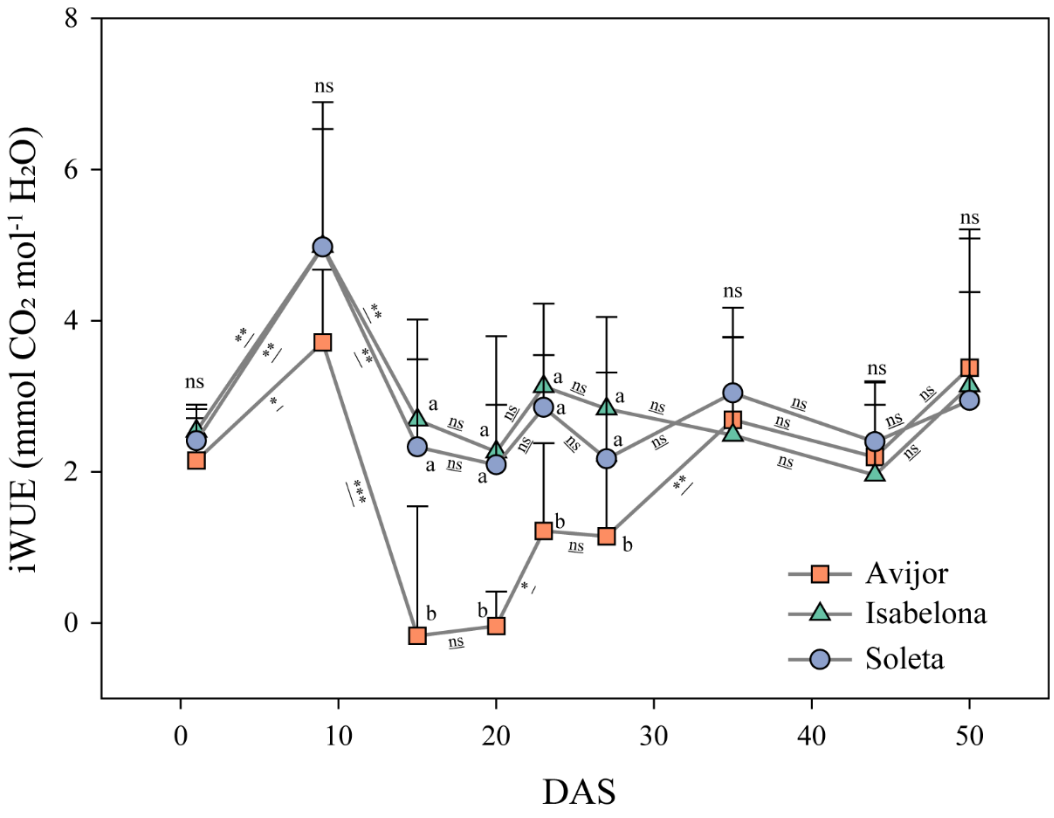

Supplementary figure 1. Instantaneous water use efficiency (iWUE) (+ standard deviation) of Avijor, Isabelona, and Soleta cultivars during the progression of the pot desiccation treatment. DAS: days after stress. Different letters indicate differences between cultivars at the same DAS according to Tukey HSD test ($p < 0.05$). Asterisks indicate significant differences among DAS within the same cultivar according to Tukey HSD test. (ns = non significant, * = significant at $p < 0.05$, ** = significant at $p < 0.01$, *** = significant at $p < 0.0001$).
